# C-Terminal Farnesylation of UCH-L1 Plays a Role in Transport of Epstein-Barr Virus Primary Oncoprotein LMP1 to Exosomes

**DOI:** 10.1128/mSphere.00030-18

**Published:** 2018-02-07

**Authors:** E. Kobayashi, M. Aga, S. Kondo, C. Whitehurst, T. Yoshizaki, J. S. Pagano, J. Shackelford

**Affiliations:** a Lineberger Comprehensive Cancer Center, University of North Carolina, Chapel Hill, North Carolina, USA; b Division of Otolaryngology–Head and Neck Surgery, Graduate School of Medicine, Kanazawa University, Takaramachi, Kanazawa, Japan; University of Pittsburgh

**Keywords:** exosome, farnesylation, metastasis, oncovirus

## Abstract

Increasing evidence shows that exosomes are key regulators in cancer cell-to-cell communication. Several reports on Epstein-Barr virus (EBV)-related malignancies demonstrate that latent membrane protein 1 (LMP1) secreted by exosomes derived from EBV- or LMP1-positive cells can promote cancer progression and metastasis. However, the mechanism by which LMP1 is loaded into exosomes is still poorly understood. Here, we examined whether the process of LMP1 loading into exosomes is linked to the multifunctional molecule of the ubiquitin system—ubiquitin C-terminal hydrolase-L1 (UCH-L1). For the first time, we demonstrate that LMP1 is physically associated with UCH-L1 and that directing of LMP1 to exosomes is mediated by C-terminal farnesylation of UCH-L1. Additionally, we found that the FTI-277 farnesyltransferase inhibitor reduces motility- and anchorage-independent growth of EBV-positive cells in functional assays. On the basis of our results, we conclude that C-terminal farnesylation of UCH-L1 is one of the key mechanisms by which LMP1 is sorted to exosomes. We hypothesize that inhibition of farnesylation with specific small-molecule inhibitors blocks exosome-mediated transfer of prometastatic molecules such as LMP1 during cancer cell-to-cell communications and thereby impedes the process of cancer invasion.

**IMPORTANCE** Exosomes are small vesicles that cells secrete into the extracellular space, and there is increasing evidence that they have pivotal roles in cell-to-cell communication in malignancy. It is reported also that EBV-associated malignant cells, including those derived from nasopharyngeal carcinoma (NPC) and B-cell lymphoma, secrete exosomes. These EBV-related exosomes may contain viral products such as latent membrane protein 1 (LMP1) and may contribute to cancer progression. The aim of this study was to investigate the mechanism by which those viral products are loaded in exosomes. In this study, we show for the first time that ubiquitin C-terminal hydrolase-L1 (UCH-L1) and its C-terminal farnesylation, a posttranslational lipid modification, contribute to this mechanism. Our results also suggest that inhibition of UCH-L1 farnesylation is a potential therapeutic target against cancer metastasis and invasion.

## Abstract (precis)

Exosomes are small vesicles that cells secrete into the extracellular space, and there is increasing evidence that they have pivotal roles in cell-to-cell communication in malignancy. It is reported also that EBV-associated malignant cells, including those derived from nasopharyngeal carcinoma (NPC) and B-cell lymphoma, secrete exosomes. These EBV-related exosomes may contain viral products such as latent membrane protein 1 (LMP1) and may contribute to cancer progression. The aim of this study was to investigate the mechanism by which those viral products are loaded in exosomes. In this study, we show for the first time that ubiquitin C-terminal hydrolase-L1 (UCH-L1) and its C-terminal farnesylation, a posttranslational lipid modification, contribute to this mechanism. Our results also suggest that inhibition of UCH-L1 farnesylation is a potential therapeutic target against cancer metastasis and invasion.

## INTRODUCTION

Nasopharyngeal carcinoma (NPC) is a highly metastatic malignancy compared with other head-and-neck carcinomas. In fact, the most common clinical symptom of NPC is a neck mass that results from cervical lymph node metastasis (1, 2). Worldwide, Epstein-Barr virus (EBV) infection has been extensively characterized as a causal or contributory risk factor in virtually all cases of NPC. EBV persists predominantly as a latent infection in NPC which is termed latency type II (1–3). Expression is restricted to certain viral genes: latent membrane protein 1 (LMP1), LMP2, EBV nuclear antigen 1 (EBNA1), EBV-encoded RNAs (EBERs), and microRNAs (miRNAs) encoded in the BamHI rightward transcript (BART) region (3, 4).

Among them, LMP1 is considered the primary EBV oncogene participating in normal cell transformation as well as in cancer metastatic progression (3, 4). We have previously identified several molecular pathways targeted by LMP1 that promote NPC invasion and metastasis (5). Prometastatic matrix metalloproteinase 1 (MMP-1) and MMP9 are upregulated by LMP1 via nuclear factor-κB (NF-κB) and activator protein-1 (AP1) transcription factors (6–9). LMP1-mediated induction of mucin 1 (10) and of the membrane cross-linker protein Ezrin participates in the early steps of cell detachment during invasion (11). LMP1-dependent expression of the transcription factors Twist (12) and Snail (13) promotes epithelial-mesenchymal transition (EMT), and LMP1 contributes to the cancer stem cell/progenitor-like phenotype of nasopharyngeal cell lines (14). In addition, LMP1-mediated signaling is involved in chromatin remodeling through AT-rich-binding protein 1 (15) and promotes angiogenesis by upregulating several key players of the process: cyclooxygenase-2 (16), hypoxia-inducible factor-1α (HIF-1α) (17, 18), and fibroblast growth factor 2 (FGF-2) (19).

Exosomes, which represent the best-explored set of extracellular vesicles derived from multivesicular bodies (MVBs), are membrane vesicles 30 to 180 nm in diameter that participate in the transfer of active transduction molecules (proteins, DNA, RNA, lipids) (20–22) to neighboring cells and also over long distances within body fluids (23, 24).

Recent studies revealed that exosomes are key regulators of cell-to-cell communications in cancer (25–28). In the context of EBV malignancies, there are increasing reports that LMP1 is secreted within exosomes produced in EBV- or LMP1-positive cells (29–35), indicating that EBV manipulates the tumor microenvironment through exosome-mediated secretion of viral oncogenes such as LMP1 (34). We have previously demonstrated that treatment of EBV-negative cells with LMP1-positive exosomes promotes epithelial-mesenchymal transition (EMT) and increases the migration and invasiveness of the treated cells (36). Moreover, LMP1 signaling increases exosome-mediated secretion of well-known cellular proinvasion factors such as FGF-2 (29) and HIF-1α (36). Identification and analysis of the content of exosomes are promising diagnostic tools for many diseases, including cancers connected to human tumor viruses, since the content depends on specific pathological conditions and treatment responses.

However, the mechanism by which cargo molecules are loaded into exosomes is still poorly understood (20, 22, 37–40). There are several reports on the mechanism of protein sorting into exosomes, and among the most extensively investigated is the endosomal sorting complex required for transport (ESCRT), which is mediated by ubiquitination of the target protein (41). At the same time, specifically in regard to the EBV oncogene LMP1, it has been reported that N-terminal ubiquitination of the protein does not alter sorting or secretion of LMP1 to exosomes, which implies that LMP1 might be targeted to exosomes through an unknown mechanism (33).

Ubiquitin C-terminal hydrolase-L1 (UCH-L1) is mainly known as a deubiquitinating enzyme (DUB), although its other activities have also been reported previously (42–44). Expression of UCH-L1 in an adult organism is restricted to the central nervous and reproductive systems, but its *de novo* expression has been reported in numerous cancers such as lung cancer (45, 46), colorectal cancer (47), bladder cancer (48), and breast cancer (49). Tumor viruses such as EBV, human papillomavirus (HPV), and Kaposi’s sarcoma-associated herpesvirus (KSHV) also induce *UCH-L1* expression during cell transformation (50–54). A substantial amount of published data has demonstrated that UCH-L1 acts as a pro-oncogenic and prometastatic molecule in cell culture and in animal model systems (46, 51, 55–60). In most cases, UCH-L1 DUB activity has been shown to play a decisive role in its pro-oncogenic functions.

Endogenous UCH-L1 can be found in virtually any cell part and organelle not only inside but also outside both normal and transformed cells, including extracellular membrane vesicles. The latest experimental data reported by a number of groups indicate that, as a multifunctional oncogenic molecule of the ubiquitin system, UCH-L1 is involved in the regulation of cellular processes responsible for transport under normal (neural and reproductive systems) and pathological (cancer development and progression) conditions. UCH-L1 regulates secretory trafficking pathways in neurons, including those involved in synaptic structures (61) and neuromuscular junctions (62). It is also associated with all major cellular systems involved in membrane trafficking in transformed cells (63, 64). Furthermore, UCH-L1 itself has been identified as a part of the molecular cargos which exosomes and membrane protrusions transfer from donor to recipient cells (65, 66).

Farnesylation represents a lipid posttranslational modification that is catalyzed by farnesyltransferase (FTase), which attaches farnesyl to the thiol group of cysteine of the CAAX motif (in which "C" is cysteine, "A" is aliphatic amino acid, and "X" is usually serine, methionine, glutamine, alanine, or threonine) in the carboxyl terminus of a protein (63, 67–69). Farnesylation is an essential process for protein-protein interactions and protein binding to cell membranes (including intracellular membrane organelles) (70).

Farnesyl transferase inhibitors (FTIs) belong to a class of experimental cancer drugs that target protein farnesyltransferases (67). Originally, the anticancer effects of inhibitors such as FTI-277 were explained as being a consequence of their ability to block the activation of the oncogenic Ras pathway through inhibition of Ras farnesylation (71–74). Later, it was suggested that even tumor cell lines that do not harbor Ras-activating mutations are sensitive to FTIs and therefore that inhibition of protein farnesylation, while not Ras specific, still has potential for cancer therapy (75, 76).

Farnesylation is also implicated in UCH-L1 function: membrane-associated UCH-L1 is farnesylated at C220 in the C terminus, and the farnesylated form of UCH-L1 has been shown to promote α-synuclein neurotoxicity (63). Instead of the conventional sequence of protein farnesylation (where the CAAX motif consists of a cysteine residue followed by two aliphatic amino acids and an end residue [X] as follows: S/M/Q/A/T) used for membrane association of small GTPase (Ras), UCH-L1 contains an atypical farnesylation sequence at its C terminus (77). Farnesylation of UCH-L1 can be downregulated by treatment with the farnesyltransferase inhibitor (FTI-277) and/or by changing Cys220 to Ser, which suggests that the Cys220 site-specific farnesylation of UCH-L1 is responsible for its membrane association (63).

In this study, we demonstrated for the first time that C-terminal farnesylation of UCH-L1 is critical for its physical association with the EBV primary oncogene LMP1. Moreover, LMP1 sorting to exosomes depends on C-terminal farnesylation of UCH-L1: point mutation abolishing C-terminal farnesylation of the protein dramatically reduces its binding to LMP1 and transfer of the LMP1/UCH-L1 complex to the exosomes. Additionally, we demonstrate that blocking of farnesylation with FTI-277 inhibitor significantly reduces cell motility and anchorage-independent growth of EBV-positive epithelial cells in functional assays. We hypothesize that the mechanism of LMP1 sorting to exosomes includes UCH-L1 C-terminal farnesylation. Considering the recently established functional role of extracellular vesicles (ECV) in cancer progression, we suggest that specific inhibition of C-terminal farnesylation of UCH-L1 may reduce invasion and metastasis of EBV-associated LMP1-positive metastatic carcinomas.

## RESULTS

### EBV primary oncoprotein LMP1 is physically associated with endogenous UCH-L1.

**FIG 1:**
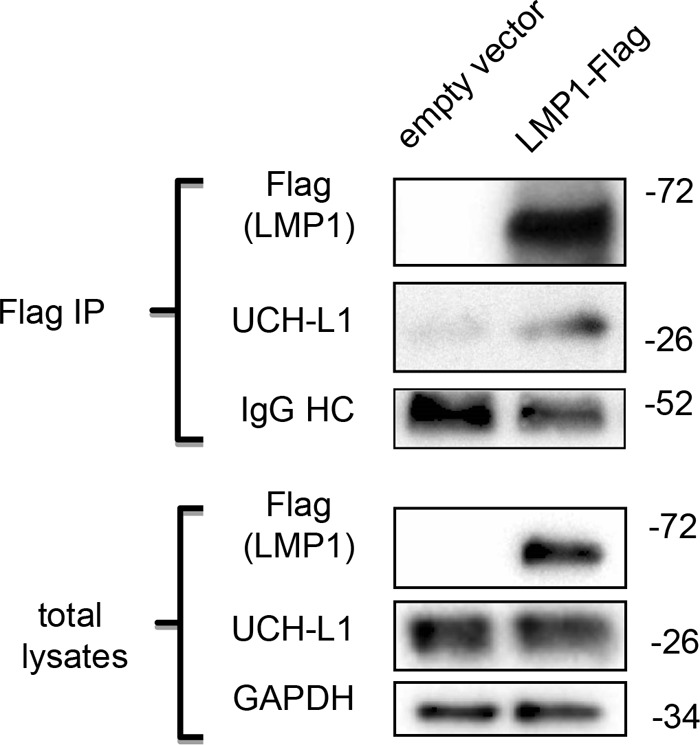
Endogenous UCH-L1 is physically associated with LMP1. 293 cells were transfected with empty and LMP1-Flag expression vectors and were harvested 48 h later for LMP1 immunoprecipitation with anti-Flag-agarose beads. IPs and total lysates of proteins were separated in 4% to 20% PAGE. After transfer to a PVDF membrane, the blots were probed with the indicated antibodies. The results shown are representative of results of three independent experiments with similar outcomes.

We previously reported that UCH-L1 expression is upregulated during EBV-mediated transformation of B-lymphocytes (52) and by expression of LMP1 in epithelial cells (50). However, whether LMP1 is directly associated with UCH-L1 was still unclear. We transfected 293 cells with a Flag-tagged LMP1 expression vector and pulled down LMP1 with anti-Flag antibody-conjugated beads. Western blotting in Fig. 1 shows the presence of endogenous UCH-L1 in LMP1 complexes but not in control immunoprecipitations (IPs) performed with anti-Flag beads after transfections performed with empty vector. This result shows that LMP1 is physically associated with endogenous UCH-L1. In addition, we observed LMP1/UCH-L1 complexes in EBV-transformed B-cell lines, and our preliminary data indicate that LMP1 C-terminal region CTAR1 is required for LMP1/UCH-L1 binding (data not shown).

### C-terminal farnesylation of UCH-L1 is required for its association with EBV oncoprotein LMP1.

**FIG 2:**
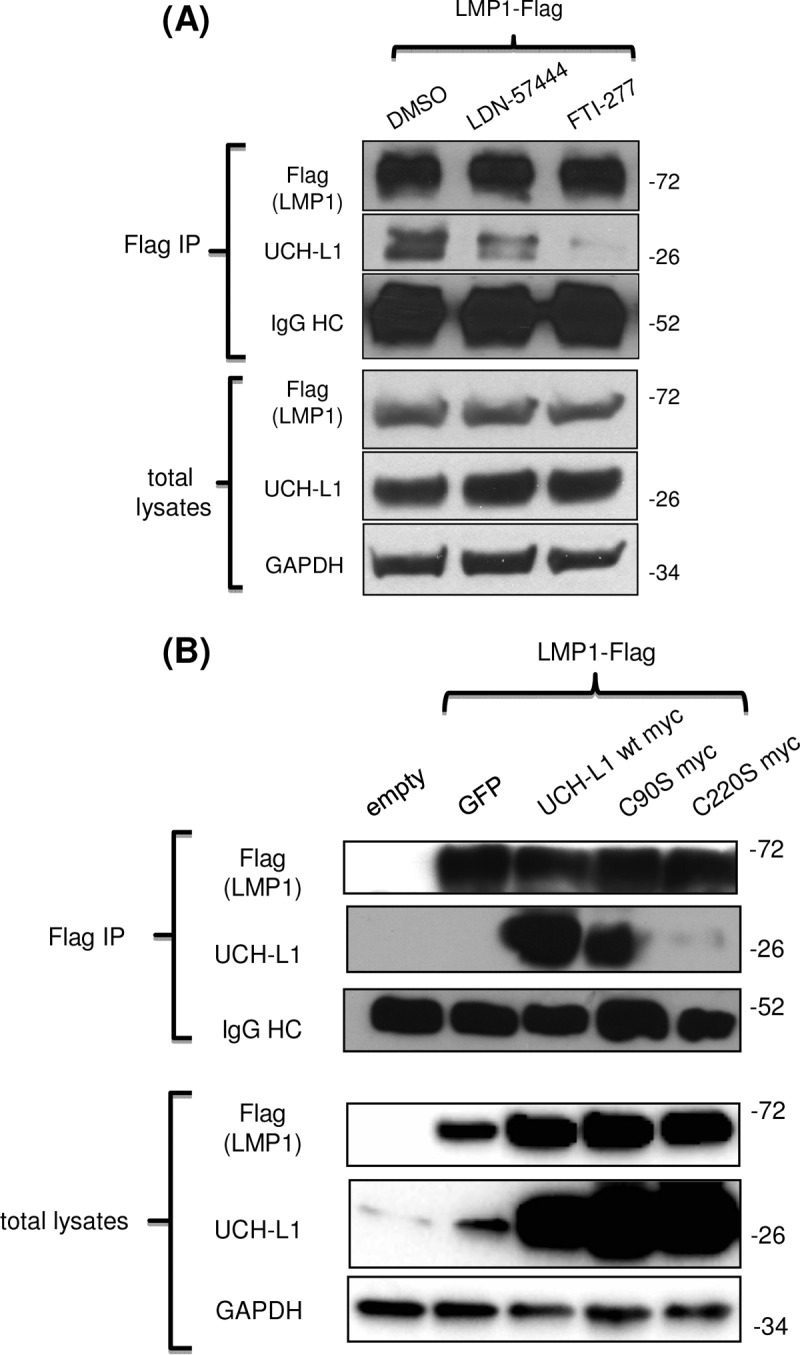
C-terminal farnesylation of UCH-L1 is required for its association with the EBV primary oncogene LMP1. (A) Inhibition of cellular farnesylation reduces LMP1 association with UCH-L1. 293 cells were transfected with empty vector, GFP (green fluorescent protein), LMP1-Flag and UCH-L1 wild-type expression vectors and treated with DMSO and either UCH-L1 DUB activity inhibitor LDN-57444 or farnesyltransferase inhibitor FTI-277 (5 μM each). At 48 h after transfection, LMP1 was immunoprecipitated with anti-Flag-agarose beads. Band intensity was quantified by the use of ImageJ (http://rsbweb.nih.gov/ij/) software. The result shows less UCH-L1 in the LMP1 complexes under conditions of treatment with FTI-277 than was seen with the DMSO control or LDN-57444 treatment. (B) Inhibition of UCH-L1-specific farnesylation inhibits LMP1/UCH-L1 complex formation. 293 cells were transfected with LMP1-Flag and the UCH-L1 wild type or one of two UCH-L1 mutants: an enzymatically inactive mutant (C90S mutant) or UCH-L1 with a mutated farnesylation site (C220S mutant). Cells were harvested 48 h posttransfection for LMP1 complex formation analysis. After IP with anti-Flag-agarose beads, Western blot analysis was performed with the indicated antibodies. The results revealed less UCH-L1 C220S mutant than wild type or C90S mutant in complex with LMP1.

Next, we examined the molecular requirements for association between LMP1 and UCH-L1. Since both LMP1 (78) and UCH-L1 (66) have been shown to be membrane-anchored cellular molecules, and since C-terminal farnesylation regulates UCH-L1 association with cellular membranes (63), we asked whether the C-terminal farnesylation of UCH-L1 is involved in the formation of complexes between LMP1 and UCH-L1. At the same time, UCH-L1 is mainly known for its deubiquitinating activity (42), so its DUB activity could affect UCH-L1 association with the EBV oncoprotein LMP1 as well. In the first set of experiments, we utilized two small-molecule inhibitors: LDN-57444 (79), a specific inhibitor of UCH-L1 deubiquitinase activity, and FTI-277 inhibitor, a selective inhibitor of cellular farnesylation of proteins (67). After transfections with Flag-LMP1 and wild-type UCH-L1, 293 cells were treated with either of two inhibitors, or with dimethyl sulfoxide (DMSO) as a control, following IP performed with anti-Flag-conjugated beads and Western blot analysis with the indicated antibodies (Fig. 2A). Quantification and normalization of UCH-L1 bands to the intensity of LMP1 bands revealed smaller amounts of LMP1-associated UCH-L1 in cells treated with FTI-277 inhibitor than in cells treated with DMSO or LDN-57444 inhibitor (Fig. 2A). This was the first indication that UCH-L1/LMP1 complex formation depends on farnesylation rather than on UCH-L1 DUB activity.

Next, we investigated whether specific UCH-L1 C-terminal farnesylation at cysteine 220 affects LMP1/UCH-L1 association. We compared the ability of wild-type UCH-L1, a DUB-dead UCH-L1 mutant (C90S mutant) (80), and a nonfarnesylated UCH-L1 mutant (cysteine 220 to serine [C220S mutant]) (63) to form cellular complexes with LMP1. After transfection of 293 cells with Flag-LMP1 and the UCH-L1 wild type or a C90S or C220S mutant, we pulled down LMP1 complexes from the cells with anti-Flag-conjugated beads. As shown in Fig. 2B, a significantly smaller amount of UCH-L1 C220S mutant was detected in LMP1 protein complexes than in those transfected with wild-type UCH-L1 or C90S DUB-dead mutant. These results demonstrate that specific C-terminal farnesylation of UCH-L1 is required for formation of its complex with LMP1. At this point, it is unclear if the farnesyl moiety is directly required for the interaction between UCH-L1 and LMP1. It is possible that the farnesyl group is necessary for localization and/or interaction with other proteins which facilitate binding of LMP1 to UCHL-1.

### LMP1 sorting to exosomes, but not to the larger extracellular vesicles, ectosomes, depends on C-terminal farnesylation of UCH-L1.

**FIG 3:**
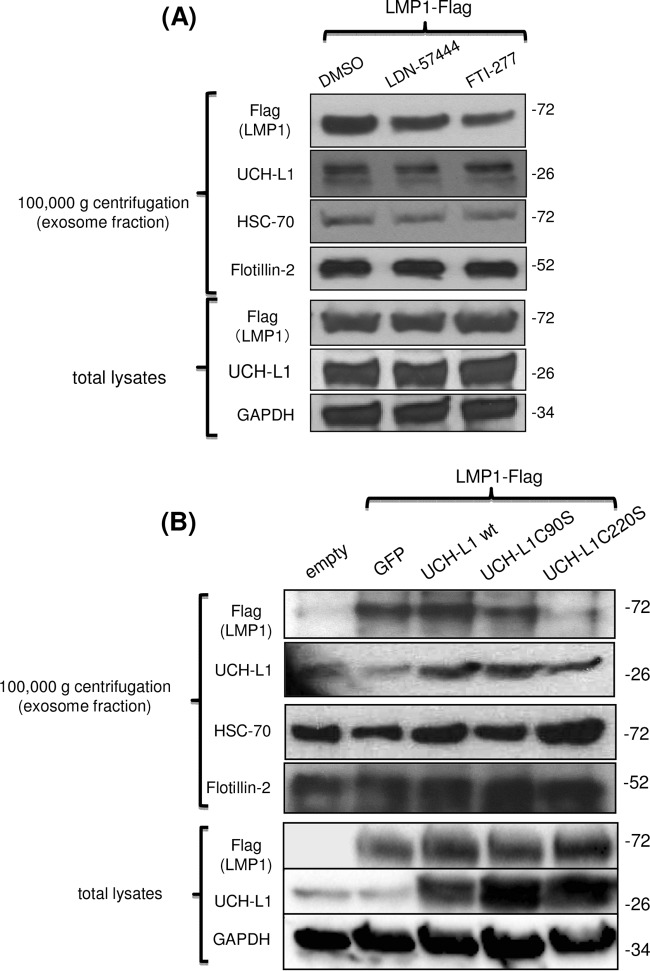
LMP1 presence in exosome fraction correlates with C-terminal farnesylation of UCH-L1. (A) Inhibition of cellular farnesylation reduces LMP1 amounts in exosomal fractions. 293 cells were transfected with LMP1 and treated with either LDN-57444 or FTI-277 inhibitors (5 μM each) or DMSO as a control. After 48 h of incubation, exosomes were purified by sequential ultracentrifugation as described in Materials and Methods. Western blot analysis demonstrates that LMP1 levels on the exosome fraction were reduced under conditions of treatment with FTI-277 compared to treatment with the DMSO control or LDN-57444 inhibitor. (B) Mutation of the farnesylation site of UCH-L1 results in reduced amounts of LMP1 in exosomes. 293 cells were transfected with LMP1 and UCH-L1 wild-type or mutant expression vectors, and exosome fractions were purified by ultracentrifugation. The results of Western blot analysis show that the LMP1 level on the exosome fraction was reduced under conditions of transfection with a farnesylation-impaired C220S mutant compared to the wild type or DUB-dead C90S UCH-L1. Protein levels in the exosomal fractions were normalized to exosome markers HSC-70 and Flotillin-2. GAPDH served as a normalization control for total lysates. GFP, green fluorescent protein.

It is becoming clear that LMP1 plays a significant role in exosome-mediated prometastatic activities in EBV-associated malignancies (81). Also, UCH-L1 has been detected in exosomal fractions of malignant cells (82). We thus investigated whether farnesylation or UCH-L1 DUB activity contributes to the sorting of LMP1 to exosomes. We first utilized LDN-57444 and FTI-277, inhibitors of UCH-L1 DUB activity and cellular farnesylation, respectively. 293 cells were transfected with LMP1 expression vector and treated with either of those two inhibitors or DMSO as a control. Exosomes were collected by differential centrifugation (fraction after centrifugation at 100,000 × *g*) from conditioned media as described previously (36). Figure 3A shows that LMP1 levels were reduced in the exosomal fraction from cells treated with FTI-277 but not in that from cells treated with LDN-57444 or DMSO.

Next, we performed similar experiments utilizing UCH-L1 C90S and C220S mutants. Transfections with wild-type UCH-L1 or DUB-dead UCH-L1 (C90S mutant) did not result in significant changes of LMP1 levels in exosomal fractions. In contrast, in the protein fractions collected from C220S UCH-L1 mutant-expressing exosomes, LMP1 levels were visibly reduced compared to those from cells transfected with LMP1 alone (Fig. 3B, top panel). This result suggests that UCH-L1 requires its C-terminal farnesylation to promote sorting of LMP1 to exosomes.

**FIG 4:**
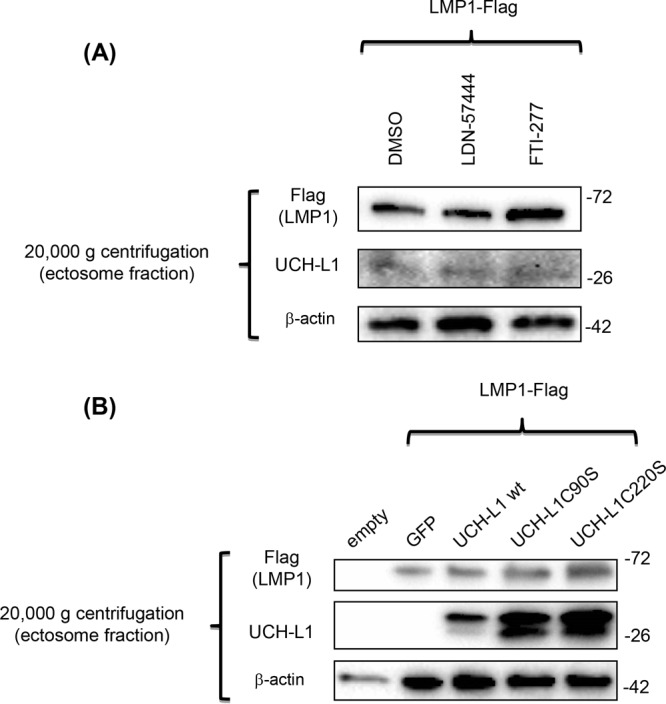
Neither C-terminal farnesylation nor deubiquitinating activity of UCH-L1 affects LMP1 presence in ectosome fractions of extracellular vesicles. The protein fractions obtained from sequential centrifugation at 20,000 × *g* (ectosomes) of the samples from the experiment represented in Fig. 3 were separated in 4% to 20% PAGE, and, after transfer to a PVDF membrane, the blots were exposed to the indicated antibodies. The results demonstrate that inhibition of cellular farnesylation with FTI-277 or UCH-L1-dependent deubiquitination with LDN-57444 (A), as well as expression of DUB-dead or farnesylation-impaired UCH-L1 mutants (B), did not change the levels of LMP1 in this fraction of extracellular vesicles.

While obtaining the exosomal fraction for the experiment whose results are shown in Fig. 3 with sequential centrifugations resulting in exosomal fractions after a final 100,000 × *g* centrifugation, we also collected a protein fraction from the same growth media after the centrifugation at 20,000 × *g* that contained larger extracellular particles, called ectosomes (83, 84). In contrast to the exosomal fraction, the levels of LMP1 were not reduced in the protein fractions of the larger particles derived from the same cells treated with the inhibitors or from the same cells expressing UCH-L1 mutants (Fig. 4). These results show that C-terminal farnesylation of UCH-L1 plays a key role in sorting of LMP1 to exosomes but not to the larger ECVs, namely, ectosomes. These results suggest that C-terminal farnesylation of UCH-L1 is a significant factor for exosome biogenesis.

### The FTI-277 inhibitor suppresses migration and anchorage-independent growth of EBV-positive epithelial cells.

**FIG 5:**
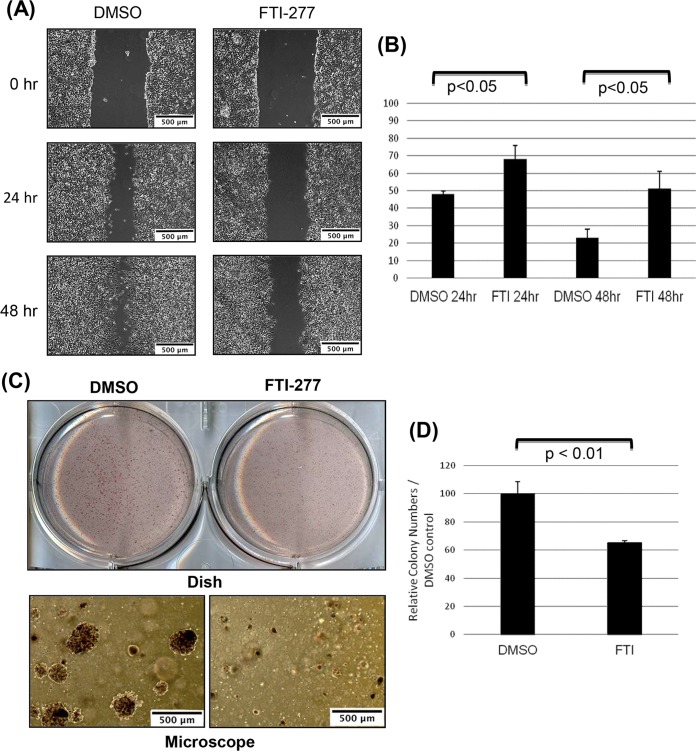
Inhibition of cellular farnesylation with FTI-277 reduces migration and colony formation of EBV-positive epithelial cells. 293EBV cells were treated with DMSO or farnesyltransferase inhibitor FTI-277 (5 μM). (A and B) Results of an *in vitro* wound-healing assay show that treatment with FTI-277 inhibits motility of EBV-positive epithelial 293 cells. Confluent monolayers of 293EBV cells were scraped with a plastic pipette tip, and migration of cells was analyzed. (A) Typical wounds examined under a microscope at 0, 24, and 48 h are shown (bars; 500 μm). (B) The widths of the “wounds” (scratched areas) at 24 and 48 h were measured by the use of ImageJ software. The percentage of the wound area was calculated by the following formula: wounded area (%) = (width after 24 h or 48 h/width at beginning) × 100%. The graph shows percentages of wound areas at 24 h and 48 h normalized to 0 h as 100% (means ± SD; *n* = 3 independent experiments [Student’s *t* test]). (C and D) The colony-forming assay was performed by seeding equal amounts of cells of different sets in soft agar in 6-well plates in triplicate. Numbers of colonies with a diameter greater than 200 μm were quantified after 10 days. (C) (Top panel) Dishes of colonies in soft agar. (Bottom panel) Colonies under the microscope. Bars, 500 μm. (D) Numbers of colonies per field were calculated. The graphs show relative colony numbers in control and FTI-277-treated dishes (means ± SD; *n* = 3 independent experiments [*t* test]).

We next examined whether inhibition of cellular farnesylation would have physiological effects on the invasive potential of EBV-positive epithelial cells. We performed two cell culture assays to evaluate motility and anchorage-independent growth of the 293 cells harboring EBV genome (85): the wound-healing assay and soft-agar colony formation assay, respectively (Fig. 5A and B). Subconfluent 293EBV cells were treated with FTI-277 inhibitor or control DMSO, scratched, and incubated for 24 h and 48 h. The result show that, compared to the control cell results, FTI-277 inhibitor suppressed migration of EBV-positive cells after both 24 h and 48 h (Fig. 5A). The distances between the wound edges were measured; graphs in Fig. 5B show that the differences in cell migration between control and FTI-treated cells were statistically significant.

Finally, we performed the soft agar colony formation assay. EBV-positive 293 cells were treated with DMSO or FTI-277 inhibitor in soft agar for 10 days, and the colonies were counted per each of 10× microscopic fields. The results in Fig. 5C show that treatment with FTI-277 reduced the ability of 293EBV cells to form colonies in soft agar and that the reduction of colony numbers was statistically significant (Fig. 5D). These results demonstrate that inhibition of cellular farnesylation suppressed anchorage-independent growth of EBV-positive epithelial cells.

## DISCUSSION

Originally, inhibitors of farnesyltransferases such as FTI-277 had been shown to reduce proliferation of many primary cancers by blocking farnesylation and, as a result, the activity of pro-oncogenic small GTPases, especially Ras (86). Moreover, several clinical trials have been carried out on FTIs with relatively promising results (71–74). Although these inhibitors were originally designed to block activation of Ras oncoprotein through its C-terminal farnesylation, this specific function might not be the sole mechanism of the antitumor activity of FTIs. In fact, the status of *ras* constitutive activation due to mutation does not correlate with FTI sensitivity (75, 76). Our results show that farnesyl transferase inhibitor FTI-277 inhibits migration and anchorage-independent growth of EBV-positive 293 cells (Fig. 5). It is important that, in the different cellular models with different levels of UCH-L1 expression, other molecular activities of this multifunctional protein might be more significant for the oncogenic phenotype. For example, we observed very modest effects of the expression of the nonfarnesylated form of UCH-L1 in NP69 cells expressing LMP1 (data not shown), probably due the still low levels of endogenous UCH-L1, especially compared to the results seen with 293 cells. The search for a specific inhibitor is necessary to evaluate of the specific role that UCH-L1 farnesylation plays in LMP1 loading into the exosomes produced by NPC tissues. At the same time, since EBV is able to infect cells of different origins, the results in 293 cells are still useful for understanding the role of UCH-L1 farnesylation in other EBV-associated carcinomas.

Recent studies demonstrated that clinically relevant low doses of one of the inhibitors, FTI-276, decreased the expression of HIF-1α and Snail (87), two essential prometastatic transcription factors induced by EBV in NP cells (13, 17). At the same time, we believe that UCH-L1 is just one of several FTI-277 targets and therefore that the mechanism of FTI-277 action is not specific for UCH-L1 and that the observed physiological inhibitory effects represent the accumulated results of FTI-277-mediated blockage of the farnesylation at several targets. For this reason, the results seen with expression of UCH-L1 C220S mutant are crucial for the claim that specific UCH-L1 farnesylation is required for UCH-L1-mediated targeting of viral oncoprotein LMP1 to exosomes (Fig. 3B).

We have previously demonstrated that HIF-1α is secreted from LMP1-positive NPC cells by exosomes and that exosomal cell-to-cell transmission of transcriptionally active HIF-1α correlated with proinvasive changes in recipient cells (36). A recent study also showed that HIF-1α activity in metastatic carcinoma *in vivo* depends on UCH-L1 (60). Considering that both LMP1 and UCH-L1 have been detected in exosomal fractions from cancer cells (30, 82), we suggested that UCH-L1 is a part of the exosomal cargo-sorting machinery and that either deubiquitinating activity or membrane-anchoring ability (C-terminal farnesylation) of UCH-L1 is involved in the process of the sorting. Ubiquitination is necessary for the most frequently reported mechanism of exosome biogenesis: the function of the endosomal sorting complex required for transport (ESCRT) (41). Recently, published work also implicated farnesylation in the process of exosome cargo loading and secretion (88).

The well-established proinvasive and prometastatic viral molecule LMP1 is a known component of exosomal cargo (30, 89). Moreover, LMP1-loaded exosomes are likely to have a critical role in modulating tumor microenvironments during invasion, contributing to the highly metastatic features of NPC and other EBV-associated malignancies (5, 90). Considering that LMP1 is a membrane protein and that UCH-L1 is associated with membranes under conditions of farnesylation, it was expected that endogenous UCH-L1 would be detected in complexes with LMP1 (Fig. 1). Furthermore, the results represented in Fig. 2 confirm that the formation of such LMP1/UCH-L1 complexes depends on the UCH-L1 site for farnesylation at the cysteine 220 residue. Interestingly, the results of these experiments also demonstrate that inhibition of UCH-L1 DUB activity with the specific LDN-57444 inhibitor (79) did not affect LMP1/UCH-L1 complex formation. It is worth mentioning that there was a certain reduction in LMP1 association with UCH-L1 after LDN-57444 treatment (Fig. 2A) and with UCH-L1 DUB-dead mutant C90S as shown in Fig. 2B, indicating that the deubiquitinating activity of UCH-L1 is involved in regulation of protein complexes, at least partially.

Nevertheless, these observations led us to the main goal of this study, that of examining a possible connection between UCH-L1 functional activities and the presence of LMP1 in exosomal fractions from EBV-positive epithelial cells. Our data show that blocking of cellular farnesylation with selective inhibitor FTI-277 and inhibition of specific C-terminal farnesylation of UCH-L1 with the use of C220S mutant inhibit LMP1 targeting to the exosomes but not its expression in the cells (Fig. 3). Blocking UCH-L1 deubiquitinating activity with the LDN-57444 inhibitor or with the overexpression of the C90S UCH-L1 DUB-dead mutant did not have a significant effect on LMP1 loading to the exosomes when the protein levels of LMP1 were normalized to the exosomal markers Flotillin and HSC-70. However, it should be noted that reduction of UCH-L1 DUB activity by the use of the LDN-57444 inhibitor or by overexpression of the DUB-dead mutant inhibited general exosome production in different cell lines (Anjali Bheda-Malge, personal observation), and we plan to investigate the role of UCH-L1 deubiquitinating activity in the exosome biogenesis in the future.

Ectosomes, with diameters ranging from 100 to 1,000 nm, represent a more heterogeneous population of microvesicles than exosomes, and they differ from exosomes in their subcellular origin as well as in the mechanism of their secretion (91). Growing evidence indicates that, along with other ECVs, ectosomes are important players in cell-to-cell communication under different conditions, including during metastatic transformation of tumor cells (92). The presence of LMP1 in ectosomes is not surprising, since it is an integral cellular membrane protein that functions as a constitutively activated member of the tumor necrosis factor receptor family. We analyzed the levels of LMP1 protein in fractions of ectosomes from the same samples of extracellular vesicles from which we obtained exosomes (see Fig. 3). Unexpectedly, in the protein fraction of these microvesicles obtained after sequential centrifugation at 20,000 × *g* (93, 94), we did not observe any changes in LMP1 levels under our experimental conditions (Fig. 4). Considering that the fractions of the exosomes reported in Fig. 3 and those of the ectosomes reported in Fig. 4 were taken from the same experiment, we concluded that UCH-L1 C-terminal farnesylation at cysteine 220 (as well as cellular farnesylation inhibited by FTI-277) regulates LMP1 targeting explicitly to exosomes. In addition, our preliminary data indicate different localizations of UCH-L1 in these two types of ECVs: during the formation of exosomes through a CD63-dependent pathway, we observed UCH-L1 in the endosomal membrane, while in the case of ectosome formation from the cellular surface membrane, UCH-L1 was instead localized inside the vesicle, along with other cargo proteins (data not shown). It is tempting to speculate that UCH-L1 plays different roles in these two distinct processes of ECV formation and that, as a deubiquitinating enzyme, UCH-L1 is an active participant in the development of multivesicular bodies (MVBs), while farnesylation of UCH-L1 is required for the loading of exosomal cargo. If this speculation is correct, the best inhibitory effect on UCH-L1 would result from the combination of two inhibitors specifically affecting both functions.

Emerging evidence reveals that cancer cells release increased amounts of different ECVs containing molecules directly stimulating invasion, metastasis, and angiogenesis (1, 95, 96). It is possible that the transfer of LMP1 itself and LMP1-induced proinvasive and proangiogenic factors might occur not only through the activity of exosomes but also through that of ectosomes. Recent studies showed that an ectosomal cargo can modulate essential processes in cancer-accosted environments in different stages of cancer progression (97). However, further investigation is needed to understand the mechanisms targeting LMP1 to different ECVs and the contribution of exosome-loaded LMP1 versus ectosome-loaded LMP1 in tumor progression.

In the present study, we examined the impact of C-terminal farnesylation of UCH-L1 not only on the formation of complexes between LMP1 and UCH-L1 but also on the targeting of LMP1 to extracellular vesicles. We propose that C-terminal farnesylation of UCH-L1 facilitates LMP1 loading in exosomes and might promote tumor invasion and metastasis through modulating the cancer microenvironment. Future investigations will show whether LMP1-loaded exosomes are not only biomarkers but also potential therapeutic targets for metastatic EBV-positive NPC. Since UCH-L1 seems to be expressed mainly in metastatic carcinomas and not in primary carcinomas, small-molecule inhibitors specifically inhibiting C-terminal farnesylation of UCH-L1 (63) might reduce the proinvasive properties of exosomes from LMP1-positive cancer cells and therefore might represent promising candidates for antimetastasis drug development.

## MATERIALS AND METHODS

### Cell culture.

Human embryonic kidney-293 (HEK-293) cells (293 cells) and EBV-expressing HEK-293 cells (293EBV cells) were used for these experiments. 293EBV cells were a gift from Wolfgang Hammerschmidt (GSF-National Research Center for Environment and Health, Munich, Germany) (98). All cells were maintained in Dulbecco’s modified Eagle medium (DMEM)–10% (vol/vol) fetal bovine serum (FBS) at 37°C in 5% CO_2_. For exosome-related assays, FBS was depleted of bovine exosomes by ultracentrifugation at 100,000 × *g* for 60 min.

### Antibodies.

Antibodies were purchased as follows: UCH-L1 (381000) from Thermo Fisher Scientific (Rockford, IL, USA); myc (sc-40) and HSC-70 (sc-7298) from Santa Cruz Biotechnology (Santa Cruz, CA, USA); Flotillin-2 (610383) from BD Biosciences (San Jose, CA); Flag (F3165) and β-actin (A1978) from Sigma-Aldrich (St. Louis, MO, USA); and GAPDH (glyceraldehyde-3-phosphate dehydrogenase; H00002597-M3) from Abnova (Taipei, Taiwan). Anti-mouse (NA931V) and anti-rabbit (NA934V) secondary antibodies for Western blotting were purchased from GE Healthcare (Little Chalfont, United Kingdom).

### Chemical agents.

Chemical agents were purchased as follows: LDN-57444 (L4170) and FTI-277 (F9803) from Sigma-Aldrich (St. Louis, MO, USA).

### Plasmids.

pcDNA3-based LMP1 has been previously described (36). Wild-type and C90S mutant UCH-L1 plasmids have been previously described (56); C220S mutant plasmid was a gift from Peter T. Lansbury, Jr. (Harvard Medical School, Cambridge, MA) (63).

### Western blotting.

Cells were lysed with buffer containing 1% sodium dodecyl sulfate (SDS) (Sigma, St. Louis, MO, USA), 50 mM Tris-HCl, 150 mM NaCl, 1 mM EDTA, and cOmplete EDTA-free protease inhibitor cocktail (11873580001; Roche Diagnostics, Mannheim, Germany). Total cell lysates were denatured in 6× Laemmli’s buffer and boiled for 5 min. Samples were separated by SDS-polyacrylamide gel electrophoresis (SDS-PAGE) and transferred to polyvinylidene fluoride (PVDF) membranes (Bio-Rad Laboratories, Hercules, CA). Membranes were blocked with a mixture of 5% milk with Tris-buffered saline–Tween 20 (TBST) and incubated overnight at 4°C with primary antibodies. Membranes were then washed with TBST and incubated with appropriate horseradish peroxidase-conjugated secondary antibodies for 1 h at room temperature. Membranes were washed again with TBST, and bands were visualized with enhanced chemiluminescence reagent (advansta, Menlo Park, CA, USA).

### Transient transfection.

293 cells were grown in 100-mm-diameter plates and transfected with 3 μg of plasmids with the use of polyethylenimine (VWR, Radnor, PA). Empty vector was used to equalize total amounts of DNA in the transfections.

### Immunoprecipitation.

Cells were lysed with buffer containing 1% Triton, 50 mM Tris-HCl, 150 mM NaCl, 1 mM EDTA, and cOmplete EDTA-free protease inhibitor cocktail (11873580001; Roche Diagnostics, Mannheim, Germany). Cell lysates were incubated with EZview Red anti-Flag M2 Affinity Gel (F2426; Sigma-Aldrich, St. Louis, MO, USA) according to the manufacturer’s instructions, and then protein was eluted from anti-Flag beads with 2× Laemmli’s buffer.

### Isolation of extracellular vesicles.

Exosomes were purified by sequential centrifugation as previously described (36). In brief, the indicated cells were grown with 10% exosome-free FBS containing DMEM, and then cell culture supernatant was collected and cell contamination removed by centrifugation at 400 × *g* for 5 min. To remove large cellular debris, the supernatant fluids were then spun at 2,000 × *g* for 10 min. Next, larger extracellular vesicles (ectosomes) were collected by centrifugation at 20,000 × *g* for 60 min. Finally, the exosome fraction was collected by centrifugation at 100,000 × *g* for 60 min. Exosomes and ectosomes were washed in phosphate-buffered saline (PBS) and pelleted again by centrifugation at the same speed.

### Wound-healing assay.

Confluent cell monolayers incubated with either FTI-277 or control DMSO were scratched with a micropipette tip, and spontaneous cell migration was monitored for 24 h and 48 h. The widths of the “wounds” (scratched areas) were measured by the use of ImageJ (http://rsbweb.nih.gov/ij/), and the healing rate of the wounds was calculated by the following formula: (width after 24 h or 48 h/width at the beginning) × 100%.

### Soft-agar colony formation assay.

Agarose was purchased from Fisher Scientific (BP165-25; Loughborough, United Kingdom). A soft-agar assay was performed by seeding 24,000 cells per well in a layer of 0.4% agar–DMEM–FBS over a layer of 1% agar–DMEM–FBS on 6-well plates. Cultures were maintained at 37°C for 10 days. Colonies were stained by the use of an iodonitrotetrazolium chloride (I8377; Sigma-Aldrich, St. Louis, MO, USA) solution, and then the colonies which had been stained by the agent and which were larger than 200 μm in diameter were counted using light microscopy.

### Statistical analysis.

Error bars in the graphical data represent means ± standard deviations (SD). Statistical significance was analyzed using Student’s *t* test. All statistical analysis was performed using EZR Software (99). A *P* value of <0.05 was considered to represent significance.
